# Supplementary material for: Synthetic Standards Combined With Error and Bias Correction Improve the Accuracy and Quantitative Resolution of Antibody Repertoire Sequencing in Human Naïve and Memory B Cells
Source: Front Immunol. 2018 Jun 20;9:1401. doi: 10.3389/fimmu.2018.01401 (PMC6019461; doi:10.3389/fimmu.2018.01401)
Supplement: Supplementary file 1 [file Presentation_1.PDF]

CDR3 amino acid sequence	CDR3 length	Variable (V) gene segment	Joining (J) gene segment	VH family	JH	IgG subclass	total mismatches	FR1 mismatches	CDR1 mismatches	FR2 mismatches	CDR2 mismatches	FR3 mismatches	spike-in pool contribution (%)
ARYANDREASY	11	IGHV3-23*01	IGHJ4*01	VH3	JH4	IgG3	0	0	0	0	0	0	7.00
ARCAFELATTE	11	IGHV3-23*01	IGHJ6*01	VH3	JH6	IgG2	9	0	4	0	5	0	0.15
ARSIMNFRIED	11	IGHV3-23*01	IGHJ1*01	VH3	JH1	IgG3	9	0	4	1	3	1	1.11
ARDRSAIREDDY	12	IGHV4-34*01	IGHJ4*01	VH4	JH4	IgG4	0	0	0	0	0	0	0.21
ARCRAIGVENTER	13	IGHV4-34*12	IGHJ6*01	VH4	JH6	IgG3	6	0	2	0	3	1	5.92
ARCHEETAHTEN	12	IGHV4-34*12	IGHJ3*01	VH4	JH3	IgG2	8	0	4	0	4	0	0.57
ARTHERESAPESCH	14	IGHV1-69*06	IGHJ4*01	VH1	JH4	IgG3	0	0	0	0	0	0	0.18
ARSSQIRREL	10	IGHV1-69*06	IGHJ6*01	VH1	JH6	IgG4	5	0	1	1	3	0	0.80
ARTARIKKHAN	11	IGHV1-69*06	IGHJ1*01	VH1	JH1	IgG3	10	0	4	0	5	1	3.04
ARCAMELLIATREE	14	IGHV1-18*01	IGHJ4*01	VH1	JH4	IgG2	0	0	0	0	0	0	0.25
ARCRISTINAPA	12	IGHV1-18*01	IGHJ6*01	VH1	JH6	IgG3	10	0	4	3	2	1	0.68
ARTETRACYCLINE	14	IGHV1-18*01	IGHJ3*01	VH1	JH3	IgG4	12	0	4	4	4	0	2.57
ARNICEECHIDNA	13	IGHV4-61*08	IGHJ4*01	VH4	JH4	IgG3	0	0	0	0	0	0	0.21
ARGIANTPANDA	12	IGHV4-61*08	IGHJ6*01	VH4	JH6	IgG2	7	0	3	0	4	0	0.94
ARGIRAFFEAFFE	13	IGHV4-61*08	IGHJ1*01	VH4	JH1	IgG3	15	0	4	3	8	0	3.04
ARIMPALAPALA	12	IGHV4-59*01	IGHJ5*01	VH4	JH5	IgG4	0	0	0	0	0	0	0.25
ARRATSEATMICE	13	IGHV4-59*01	IGHJ6*01	VH4	JH6	IgG3	10	0	4	0	4	2	0.48
ARRATTLESNAKE	13	IGHV4-59*01	IGHJ3*01	VH4	JH3	IgG2	10	1	5	0	3	1	2.17
ARREINDEER	10	IGHV3-30*03	IGHJ4*01	VH3	JH4	IgG3	0	0	0	0	0	0	0.18
ARSALAMANDER	12	IGHV3-30*03	IGHJ6*01	VH3	JH6	IgG4	9	0	3	2	4	0	1.32
ARTIGERSEVEN	12	IGHV3-30*03	IGHJ1*01	VH3	JH1	IgG3	12	0	4	0	5	3	2.17
ARTREESHREW	11	IGHV4-39*01	IGHJ4*01	VH4	JH4	IgG2	0	0	0	0	0	0	0.29
ARMANHATTAN	11	IGHV4-39*01	IGHJ6*01	VH4	JH6	IgG3	5	0	1	0	4	0	0.94
ARIRISHCAFE	11	IGHV4-39*01	IGHJ3*01	VH4	JH3	IgG4	11	2	3	2	4	0	1.84
ARNEWWHISKEY	12	IGHV3-48*02	IGHJ4*01	VH3	JH4	IgG3	0	0	0	0	0	0	0.35
ARSWEETWINE	11	IGHV3-48*02	IGHJ6*01	VH3	JH6	IgG2	7	0	3	0	4	0	1.56
ARGINMADRAS	11	IGHV3-48*02	IGHJ2*01	VH3	JH2	IgG3	11	0	4	1	6	0	1.84
ARAPPLETINIS	12	IGHV1-3*02	IGHJ4*01	VH1	JH4	IgG4	0	0	0	0	0	0	0.35
ARCAIPIRINHA	12	IGHV1-3*02	IGHJ4*01	VH1	JH4	IgG3	6	0	2	0	4	0	1.32
ARNECTARINE	11	IGHV3-21*01	IGHJ1*01	VH3	JH1	IgG2	0	0	0	0	0	0	0.41
ARPHYSALIS	10	IGHV3-21*01	IGHJ2*01	VH3	JH2	IgG3	10	0	5	0	3	2	0.80
ARAAMARANTH	11	IGHV1-2*02	IGHJ6*01	VH1	JH6	IgG4	0	0	0	0	0	0	0.57
ARGRAPESALAD	12	IGHV1-2*02	IGHJ4*01	VH1	JH4	IgG3	11	0	7	0	3	1	0.21
ARFIDDLEHEAD	12	IGHV4-31*02	IGHJ4*01	VH4	JH4	IgG2	0	0	0	0	0	0	0.48
ARFRIEDGARLIC	13	IGHV4-31*02	IGHJ1*01	VH4	JH1	IgG3	8	0	2	0	6	0	0.15
ARFATCHICKEN	12	IGHV3-33*01	IGHJ2*01	VH3	JH2	IgG4	0	0	0	0	0	0	0.48
ARDRIEDSEAWEED	14	IGHV3-33*01	IGHJ6*01	VH3	JH6	IgG3	8	0	3	0	1	4	1.56
ARSWISSCHARD	12	IGHV5-51*01	IGHJ4*01	VH5	JH4	IgG2	0	0	0	0	0	0	0.29
ARWATERCRESS	12	IGHV5-51*01	IGHJ4*01	VH5	JH4	IgG3	2	0	2	0	0	0	1.11
AREGGPLANT	10	IGHV1-46*01	IGHJ1*01	VH1	JH1	IgG4	0	0	0	0	0	0	0.41
ARARGENTINA	11	IGHV1-46*01	IGHJ6*01	VH1	JH6	IgG3	5	0	3	0	1	1	0.18
ARAEINSTEIN	11	IGHV3-7*01	IGHJ4*01	VH3	JH4	IgG2	0	0	0	0	0	0	0.29
ARKASACHSTAN	12	IGHV3-7*01	IGHJ4*01	VH3	JH4	IgG3	8	0	4	0	4	0	0.94
ARAPICCARD	10	IGHV4-38-2*02	IGHJ1*01	VH4	JH1	IgG2	0	0	0	0	0	0	0.25
ARNETHERLANDS	13	IGHV4-38-2*02	IGHJ6*01	VH4	JH6	IgG3	6	0	1	1	4	0	0.80
ARSELFIESTICK	13	IGHV3-11*01	IGHJ4*01	VH3	JH4	IgG2	0	0	0	0	0	0	0.41
ARPEKINGENTE	12	IGHV3-11*01	IGHJ3*01	VH3	JH3	IgG3	6	0	3	1	2	0	1.32
ARCHAIMASALA	12	IGHV1-8*01	IGHJ1*01	VH1	JH1	IgG2	2	0	0	2	0	0	0.21
ARGINGERALE	11	IGHV1-8*01	IGHJ6*01	VH1	JH6	IgG3	6	1	0	4	1	0	0.68
ARGREATREDMEAT	14	IGHV3-66*03	IGHJ5*01	VH3	JH5	IgG2	0	0	0	0	0	0	0.68
ARRACLETTE	10	IGHV3-66*03	IGHJ3*01	VH3	JH3	IgG3	15	0	3	2	6	4	0.25
ARAMYSMART	10	IGHV3-30*04	IGHJ1*01	VH3	JH1	IgG2	0	0	0	0	0	0	0.29
ARENDLESSWAY	12	IGHV3-30*04	IGHJ6*01	VH3	JH6	IgG3	11	1	4	4	2	0	0.80
ARLANADELREY	12	IGHV2-5*01	IGHJ4*01	VH2	JH4	IgG2	0	0	0	0	0	0	4.24
ARCDELEVINGNE	13	IGHV2-5*01	IGHJ3*01	VH2	JH3	IgG3	3	0	3	0	0	0	1.11
ARTHEISLAND	11	IGHV3-64*02	IGHJ2*01	VH3	JH2	IgG3	0	0	0	0	0	0	3.59
ARRFEDERER	10	IGHV3-64*02	IGHJ6*01	VH3	JH6	IgG3	8	0	4	1	3	0	0.57
ARVANILLACAKE	13	IGHV4-4*07	IGHJ4*01	VH4	JH4	IgG3	0	0	0	0	0	0	0.35
ARWALLSTREET	12	IGHV4-4*07	IGHJ3*01	VH4	JH3	IgG3	7	0	2	0	5	0	1.11
ARGRAYALDER	11	IGHV3-15*01	IGHJ2*01	VH3	JH2	IgG3	0	0	0	0	0	0	1.84
ARCHERRYPIE	11	IGHV3-15*01	IGHJ6*01	VH3	JH6	IgG3	4	0	0	2	2	0	0.25
ARSPICYSHRIMP	13	IGHV2-26*01	IGHJ5*01	VH2	JH5	IgG3	0	0	0	0	0	0	0.68
ARFRESHTHYME	12	IGHV2-26*01	IGHJ3*01	VH2	JH3	IgG3	6	0	4	0	2	0	0.41
ARTRAVELLING	12	IGHV3-74*01	IGHJ2*01	VH3	JH2	IgG3	0	0	0	0	0	0	0.94
ARFMAGELLAN	11	IGHV3-74*01	IGHJ6*01	VH3	JH6	IgG3	7	0	1	2	4	0	0.35
ARSWISSNESS	11	IGHV2-70*13	IGHJ1*01	VH2	JH1	IgG3	0	0	0	0	0	0	0.15
ARMILKSHAKE	11	IGHV4-30-4*01	IGHJ2*01	VH4	JH2	IgG3	0	0	0	0	0	0	0.48
ARGREENPEPPER	13	IGHV7-4-1*02	IGHJ4*01	VH7	JH4	IgG3	0	0	0	0	0	0	0.57
ARANGELFALLS	12	IGHV3-53*01	IGHJ6*01	VH3	JH6	IgG3	0	0	0	0	0	0	0.29
ARDEATHVALLEY	13	IGHV6-1*01	IGHJ1*01	VH6	JH1	IgG3	0	0	0	0	0	0	0.41
ARVARANASI	10	IGHV4-30-2*03	IGHJ3*02	VH4	JH3	IgG3	0	0	0	0	0	0	0.35
ARSTARWARS	10	IGHV4-28*01	IGHJ4*01	VH4	JH4	IgG3	0	0	0	0	0	0	4.24
ARFISHNCHIPS	12	IGHV3-9*01	IGHJ6*01	VH3	JH6	IgG3	0	0	0	0	0	0	5.92
ARITSATEST	10	IGHV3-20*01	IGHJ2*01	VH3	JH2	IgG3	0	0	0	0	0	0	2.57
ARHERAKLES	10	IGHV1-24*01	IGHJ3*01	VH1	JH3	IgG3	0	0	0	0	0	0	4.24
ARWILLIAMKELT	13	IGHV3-49*03	IGHJ5*01	VH3	JH5	IgG3	0	0	0	0	0	0	3.04
ARMINERVATHECAT	15	IGHV1-69-2*01	IGHJ6*01	VH1	JH6	IgG3	2	0	0	0	2	0	1.56
ARTARDIGRADA	9	IGHV5-10-1*02	IGHJ2*01	VH5	JH2	IgG3	0	0	0	0	0	0	1.32
ARRFRANKLIN	11	IGHV1-58*02	IGHJ3*01	VH1	JH3	IgG3	0	0	0	0	0	0	0.80
ARSELACHII	10	IGHV3-72*01	IGHJ4*01	VH3	JH4	IgG3	0	0	0	0	0	0	0.57
ARTEHRANINIRAN	14	IGHV3-73*02	IGHJ6*01	VH3	JH6	IgG3	0	0	0	0	0	0	0.18
ARDAMAVAND	10	IGHV3-13*01	IGHJ2*01	VH3	JH2	IgG3	0	0	0	0	0	0	0.21
ARNEMERTEA	10	IGHV1-45*02	IGHJ4*01	VH1	JH4	IgG3	0	0	0	0	0	0	0.48
ARKANAMYCIN	11	IGHV3-43*01	IGHJ4*01	VH3	JH4	IgG3	0	0	0	0	0	0	0.68
ARTRAGGIAI	10	IGHV7-81*01	IGHJ6*01	VH7	JH6	IgG3	0	0	0	0	0	0	0.48

Supplementary table 1. Molecular characteristics of 85 synthetic human IgH gene standards ('spike-ins') used in this study. See Figure 1A for spike-in construction schematic.

Sample	Total RNA extracted (ng/uL)	Total RNA (total amount)	Average number of cDNA transcripts per uL	Total number of cDNA transcripts	Sequencing depth (number of reads prior to pre-procssing)	Number of reads after pre-processing
IgM1_D1	6.6	132	5'109	62'581	676'557	403'271
IgM2_D1	59.1	1182	4'691	57'469	781'452	508'998
IgM3_D1	10.1	202	3'503	42'916	2'236'338	1'039'415
IgM4_D1	30.6	612	405	4'955	792'330	437'560
IgM5_D1	19	380	3'293	40'333	1'519'422	529'933
IgM1_D2	3.7	74	3'390	41'528	1'925'305	1'111'250
IgM2_D2	53.3	1066	4'330	53'043	673'482	379'811
IgM3_D2	34.4	688	2'048	25'088	992'456	488'230
IgM4_D2	2.2	44	3'075	37'669	981'274	452'817
IgM5_D2	47.4	948	3'855	47'220	2'279'624	440'083
IgM1_D3	7.7	154	4'631	56'734	545'947	333'492
IgM2_D3	29.5	590	3'568	43'702	1'448'340	636'432
IgM3_D3	2.6	52	2'860	35'035	649'626	407'914
IgM4_D3	7.2	144	3'567	43'696	986'419	529'860
IgG1_D1	61.2	1224	1'277	15'643	4'282'524	1'005'440
IgG2_D1	8.7	174	1'289	15'784	2'513'370	2'099'114
IgG3_D1	12.1	242	1'549	18'969	720'804	635'650
IgG4_D1	9.4	188	1'577	19'312	1'057'258	919'252
IgG5_D1	30.7	614	2'884	35'325	1'497'219	1'306'234
IgG1_D2	52.4	1048	421	5'157	1'049'571	913'558
IgG2_D2	3.7	74	181	2'211	959'085	700'808
IgG3_D2	8.5	170	268	3'277	910'336	793'379
IgG4_D2	6.5	130	214	2'622	747'740	662'657
IgG5_D2	5.6	112	386	4'722	998'038	826'427
IgG1_D3	7	140	1'552	19'012	604'835	490'991
IgG2_D3	5.4	108	683	8'367	804'122	658'452
IgG3_D3	5.9	118	972	11'901	756'943	649'224
IgG4_D3	6.9	138	1'639	20'072	2'724'771	1'179'968

Supplementary table 2. Overview over our experimental results

Sample	Aligned reads (consensus build)	Raw CDR3 variants (AA)	Raw CDR3 variants w.o. singletons (AA)	Raw total variants (whole VDJ, nt)	MAF corrected CDR3 variants (AA)	MAF corrected clonotypes (Same V/J Gene, same	MAF corrected variants (whole VDJ, nt)	Donor
IgM1_D1	201'889	40'523	19'811	109'400	14'932	14'617	18'226	D1
IgM2_D1	260'867	37'896	14'948	129'737	11'276	11'079	13'695	D1
IgM3_D1	393'067	47'139	17'310	155'812	11'745	11'604	14'109	D1
IgM4_D1	203'127	45'901	21'113	128'923	15'147	14'883	18'036	D1
IgM5_D1	175'215	32'291	14'867	96'267	11'402	11'172	14'175	D1
IgM1_D2	534'227	65'691	24'239	234'409	18'123	17'643	23'793	D2
IgM2_D2	214'469	37'763	16'138	117'586	12'277	12'055	14'635	D2
IgM3_D2	244'601	35'646	14'290	120'036	10'668	10'443	13'381	D2
IgM4_D2	204'029	37'596	16'694	111'370	12'521	12'282	15'338	D2
IgM5_D2	135'195	26'179	12'551	66'864	9'406	9'292	10'827	D2
IgM1_D3	179'806	32'363	12'975	97'508	9'970	9'798	12'564	D3
IgM2_D3	285'749	42'877	18'423	138'159	14'127	13'862	17'709	D3
IgM3_D3	197'051	33'642	15'522	101'694	11'778	11'571	14'547	D3
IgM4_D3	248'200	31'440	12'497	103'177	9'700	9'575	11'836	D3
IgG1_D1	207'903	32'327	9'110	82'511	7'177	6'637	9'034	D1
IgG2_D1	1'484'045	159'340	36'815	521'274	8'555	7'612	13'519	D1
IgG3_D1	489'704	83'577	19'244	246'707	11'000	9'754	15'149	D1
IgG4_D1	712'466	104'829	24'906	322'761	11'346	10'075	17'800	D1
IgG5_D1	1'025'422	162'032	38'430	489'885	18'735	16'137	29'100	D1
IgG1_D2	644'950	56'659	13'354	199'190	3'246	2'984	5'023	D2
IgG2_D2	429'902	24'931	6'379	81'182	1'657	1'447	2'803	D2
IgG3_D2	541'960	38'113	8'976	134'687	1'975	1'836	2'937	D2
IgG4_D2	472'428	25'162	5'940	83'522	1'402	1'283	2'063	D2
IgG5_D2	566'689	39'991	9'372	132'272	2'507	2'308	3'641	D2
IgG1_D3	367'556	74'016	16'686	206'957	10'674	9'649	14'984	D3
IgG2_D3	485'151	55'056	12'786	182'378	4'557	4'191	6'376	D3
IgG3_D3	502'248	60'102	14'118	206'762	5'439	5'028	7'768	D3
IgG4_D3	693'622	95'787	22'388	325'068	9'264	8'439	14'210	D3

Supplementary table 2. Overview over our experimental results

Primer	Sequence	Notes
IgG_1r	TTGGCACCCGAGAATTCCACTGHHHHHACAHHHHHACAHHHHNATTGTTCTGGGAAGTAGTCCTTGACCAG	Red part indicates primer binding site, black part contains unique identifier and overhang
IgM_1r	TTGGCACCCGAGAATTCCACTGHHHHHACAHHHHHACAHHHHNATTACGAGGGGGAAAAGGGTTGG	see above
VH1a	CGTTCAGAGTTCTACAGTCCGACGATCHHHHACHHHHACHHHNGCAGGCAGCTGGTGCAGTCTGGGG	see above
VH1b	CGTTCAGAGTTCTACAGTCCGACGATCHHHHACHHHHACHHHNGCAGTCAGCTGGTGCAGTCTGGAG	see above
VH1c	CGTTCAGAGTTCTACAGTCCGACGATCHHHHACHHHHACHHHNGCAGCCAGCTTGTGCAGTCTGGGG	see above
VH1d	CGTTCAGAGTTCTACAGTCCGACGATCHHHHACHHHHACHHHNGCAGGCAGCTGGTGCAGTCTGGGC	see above
VH2a	CGTTCAGAGTTCTACAGTCCGACGATCHHHHACHHHHACHHHNGCAGCCCAGGTCACCTTGAAGGAGTCTG	see above
VH2b	CGTTCAGAGTTCTACAGTCCGACGATCHHHHACHHHHACHHHNGCAGCCCAGATCACCTTGAAGGAGTCTG	see above
VH3a	CGTTCAGAGTTCTACAGTCCGACGATCHHHHACHHHHACHHHNGCAGTGTGAGGTGCAGCTGGTGGAGTC	see above
VH3b	CGTTCAGAGTTCTACAGTCCGACGATCHHHHACHHHHACHHHNGCAGTGTGAAGTGCAGCTGGTGGAGTC	see above
VH3c	CGTTCAGAGTTCTACAGTCCGACGATCHHHHACHHHHACHHHNGCAGTGTCAGGTGCAGCTGGTGGAGTC	see above
VH3d	CGTTCAGAGTTCTACAGTCCGACGATCHHHHACHHHHACHHHNGCAGTGTGAGGTGCAGCTGGTGGAGAC	see above
VH4a	CGTTCAGAGTTCTACAGTCCGACGATCHHHHACHHHHACHHHNGCAGTGCAGCTGCAGGAGTCGGG	see above
VH4b	CGTTCAGAGTTCTACAGTCCGACGATCHHHHACHHHHACHHHNGCAGTACAGCTGCAGGAGTCGGG	see above
VH4c	CGTTCAGAGTTCTACAGTCCGACGATCHHHHACHHHHACHHHNGCAGTGCAGCTGCAGGAGTCCGG	see above
VH4d	CGTTCAGAGTTCTACAGTCCGACGATCHHHHACHHHHACHHHNGCAGTGCAGCTACAGCAGTGGGG	see above
VH5	CGTTCAGAGTTCTACAGTCCGACGATCHHHHACHHHHACHHHNGCAGGCAGCTGGTGCAGTCTGGAG	see above
VH6	CGTTCAGAGTTCTACAGTCCGACGATCHHHHACHHHHACHHHNGCAGTACAGCTGCAGCAGTCAGG	see above
VH7	CGTTCAGAGTTCTACAGTCCGACGATCHHHHACHHHHACHHHNGCAGTGCAGCTGGTGCAATCTGG	see above
PCR1_1r	ACTGGAGTTCCTTGGCACCCGAGAATTCCACT*G	'_*' indicates phosphorothioate bond
PCR2_f	AATGATACGGCGACCACCGAGATCTACACGTTCAGAGTTCTACAGTCCGACGAT*C	'_*' indicates phosphorothioate bond
PCR2_r	CAAGCAGAAGACGGCATACGAGATXXXXXXGTGACTGGAGTTCCTTGGCACCC	Red X indicates Illumina index
IgG_probe	/56-FAM/T+CTT+CCCC+C+TG+G/3IABkFQ/	'+_' indicate locked ribonucleic acids, '/56'-FAM = 5' 6-FAM (Fluorescein), '/3IABkFQ/' = 3' Iowa Black FQ
IgM_probe	/56-FAM/C+CCC+AA+CC+C+TTT/3IABkFQ/	'+_' indicate locked ribonucleic acids, '/56'-FAM = 5' 6-FAM (Fluorescein), '/3IABkFQ/' = 3' Iowa Black FQ
Spike_probe	/5HEX/CG+T C+T+G ACT +AGA +ACT +C/3IABkFQ/	'+_' indicate locked ribonucleic acids, '/56'-FAM = 5' HEX (Hexachlorofluorescein),'/3IABkFQ/' = 3' Iowa Black FQ
ddPCR_f	GGTCACYGTCTCYTCAG	
ddPCR_r	TGGCACCCGAGAATTC	

Supplementary table 3. Overview over all primers and probes used in this study

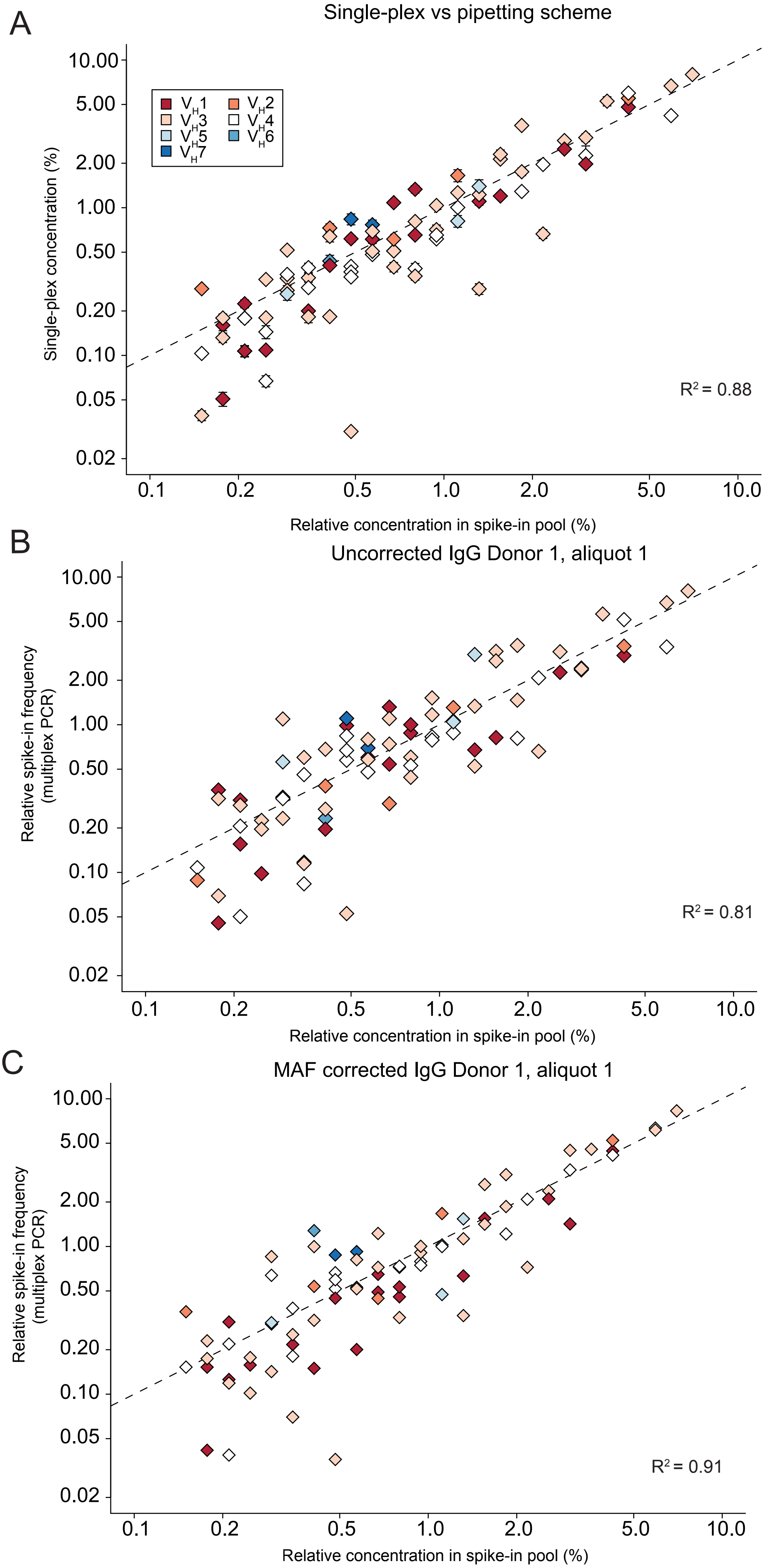

Figure S1. The sequencing bias of the multiplexed primer set was assessed by plotting the measured frequencies of each standard versus its actual, pipetted concentration in the pool. In an ideal case, the measured frequencies would fall onto the dashed line. The deviation from this line was used to calculate the R2 value, which decreases with greater deviation. (A) The upper panel shows the divergence of the measured frequencies obtained in the singleplex experiments versus pipetted concentration. Panels (B) and (C) show the measured frequencies and their deviations for one single experiment (IgG, Donor 1, Aliquot 1) before and after MAF correction.

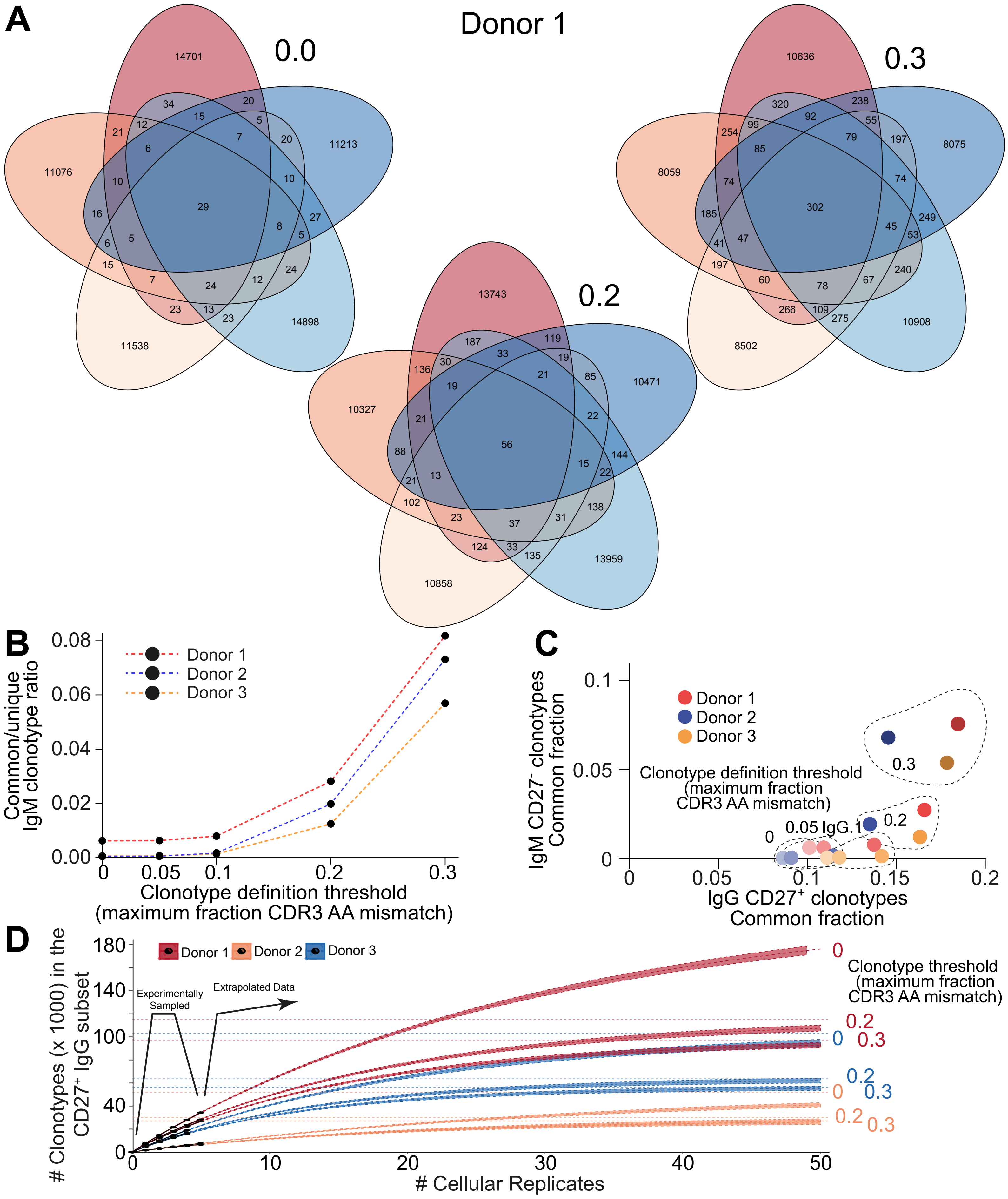

Supplemental Figure 2. Clonotype definition stringency does not create analysis bias comparing naive to memory BCR repetoires. (A) Venn diagrams showing the presence of clonotypes shared across cellular replicates (2 x 10^5 cells each) from the naive ($CD27^-$ IgM) B cell subset of donor 1. Sequence membership in a clonotype was defined by common IGHV and IGHJ usage, as well as CDR3 amino acid sequence deviation no greater than (from left to right plots) 0%, 20%, or 30%, respectively, from the clonotype consensus sequence. (B) Ratio of IgM clonotypes common to multiple cellular aliquots (overlapping regions from Venn Diagrams in panel (A) to clonotypes found only in a single cellular sample as CDR3 conservation stringency is relaxed during clustering. (C) Correlation between common (found in multiple cellular aliquots) and unique (found in only one cellular aliquot) clonotypes for $CD27^-$ IgM and $CD27^+$ IgG subsets per donor as CDR3 conservation criteria for clonotype membership of a sequence are relaxed. (D) Estimation of $CD27^+$ IgG BCR clonotype repertoire size in the entire peripheral blood volume given variable thresholds.

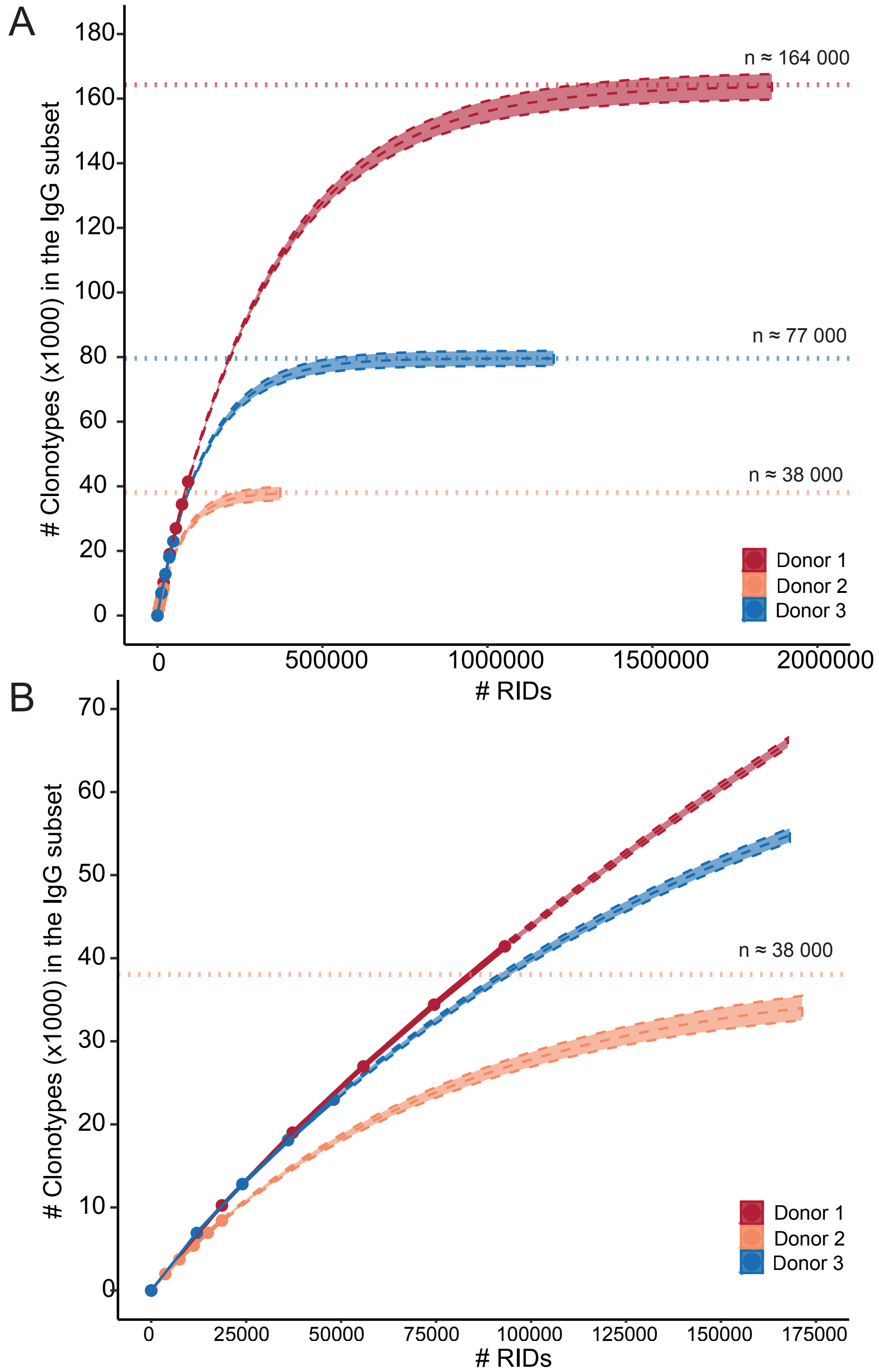

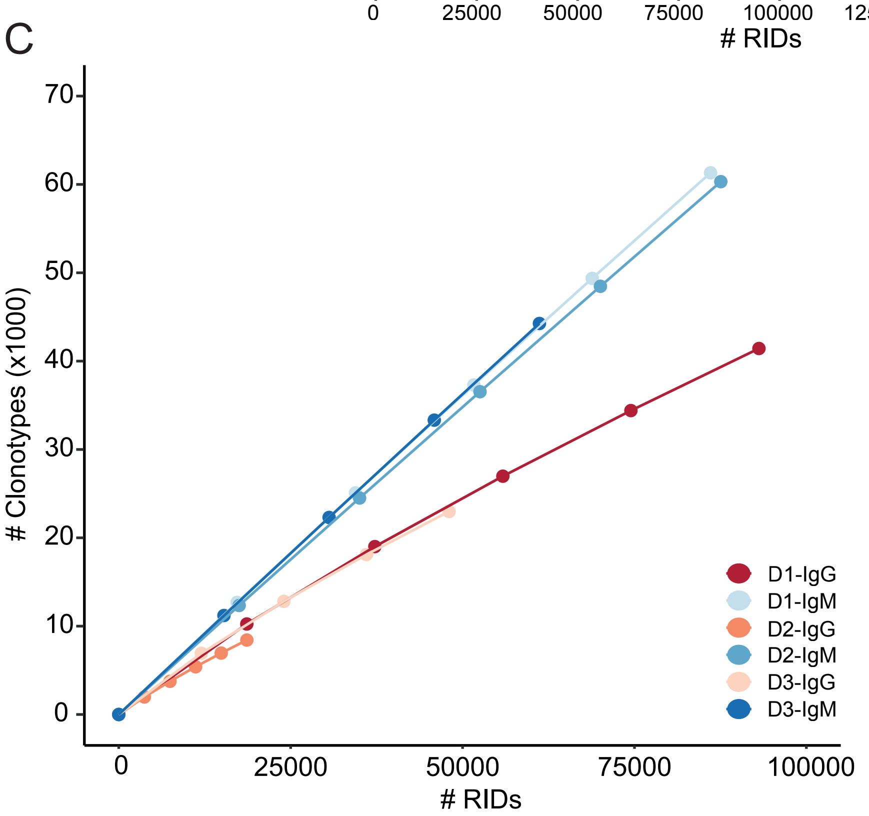

Figure S3. (**A**) Repeatedly observed sequences within the first 5 cellular aliquots allow determination of the number of distinct clonotypes in the IgG CD27+ subset (Chao2 estimator, D1 = 164 268 ± 2 365, D2 = 38 034 ± 1 302, D3 = 76 904 ± 1 409), as well as the approximate amount of cDNA transcripts / RIDs required to discover all clonotypes. (**B**) Expanded view of panel A. (**C**) The average number of newly discovered clonotypes for each additional replicate (for both the IgM and the IgG subset).

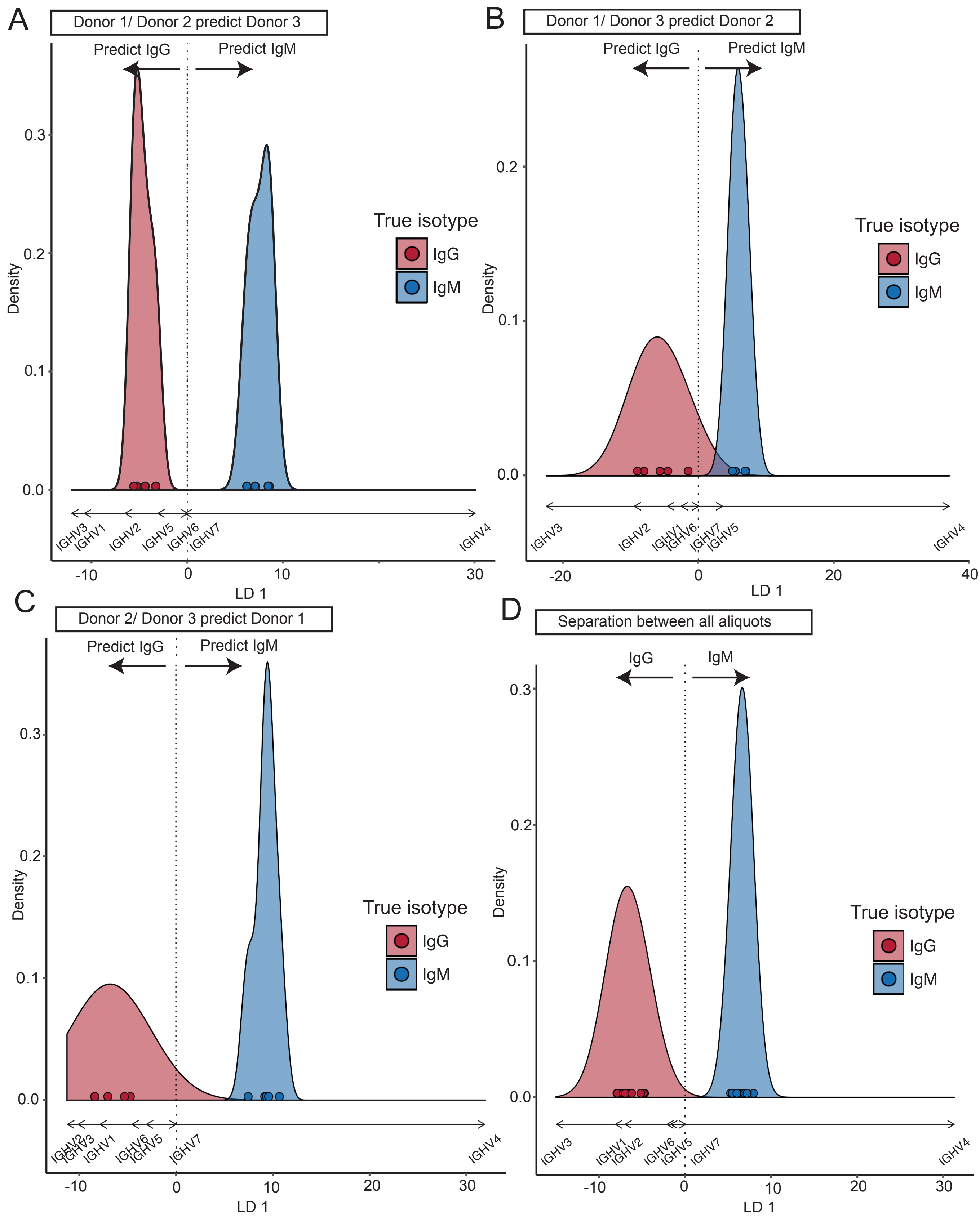

Fig. S4. Linear discriminant analysis distinguishes CD27+ IgG repertoires and CD27- IgM repertoires based on their V-Gene family usage. (**A**) All aliquots from donors 1 and 2 were used to fit an LDA classifier based on the centered log ratio transformed V-Gene family frequencies. Afterwards, the aliquots from donor 3 are projected to the fitted component axis. Positive and negative values predict IgM and IgG repertoires, respectively. Arrows below the plot indicate the contribution of each V-Gene family to the prediction. Colored dots show true class membership and their positions are smoothed using kernel desnity estimators. Panels (**B**-**D**) shows the same procedure for different splits of the data.

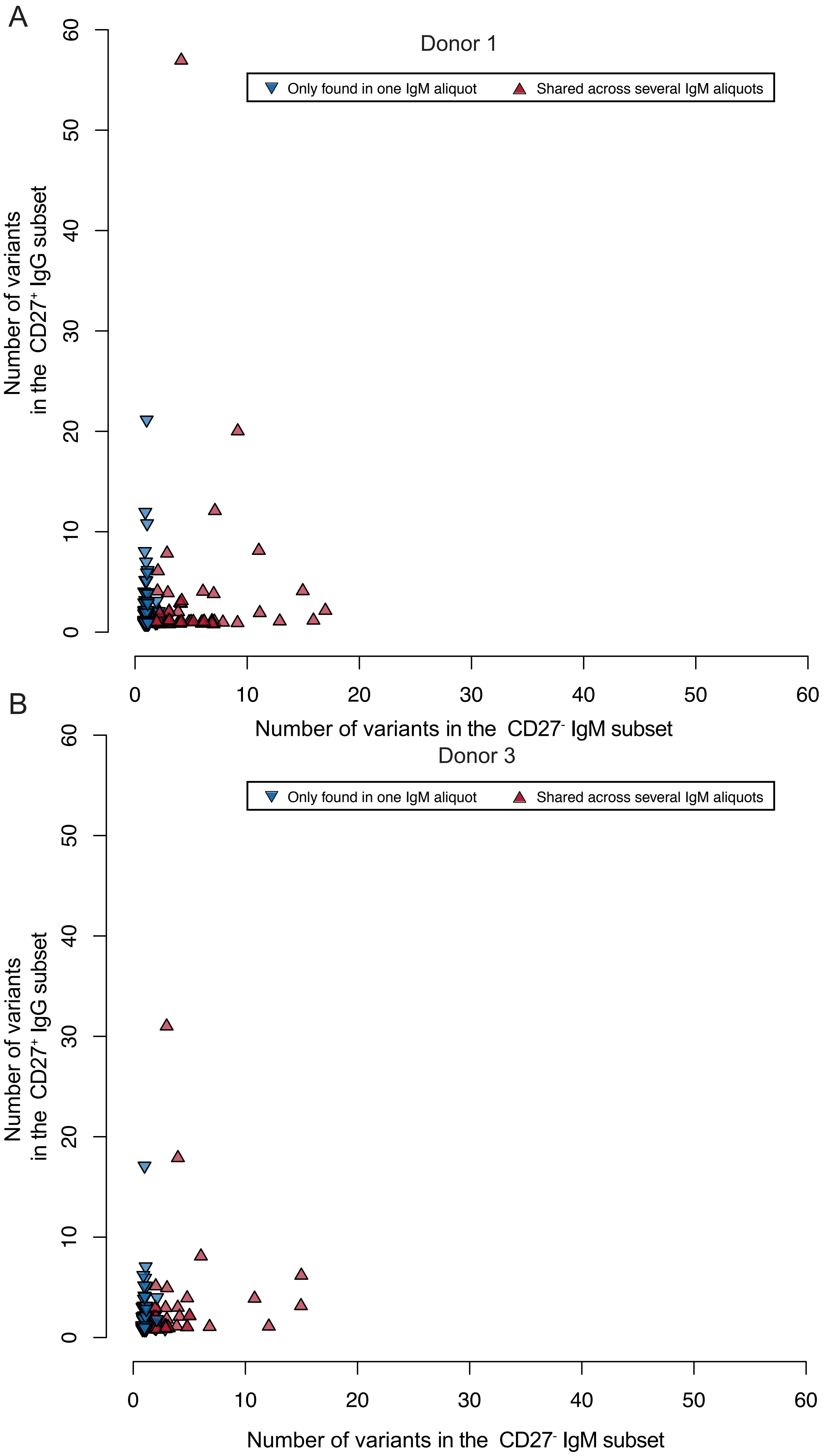

Figure S5. Clonal composition of each clonotype that is shared between the IgG and IgM subsets in terms ofits IgG and IgM variants. The red, upward pointing triangle indicates clonotypes that are expaned in the IgM repertoire, whereas the blue triangle highlights clonotypes which could only be found in one IgM aliquot. Panels (**A**) and (**B**) show the results for donors 1 and 3, respectively.
